# The Rubber Hand Illusion: Feeling of Ownership and Proprioceptive Drift Do Not Go Hand in Hand

**DOI:** 10.1371/journal.pone.0021659

**Published:** 2011-06-28

**Authors:** Marieke Rohde, Massimiliano Di Luca, Marc O. Ernst

**Affiliations:** 1 Department of Cognitive Neuroscience, University of Bielefeld, Bielefeld, Germany; 2 Multisensory Perception and Action Group, Max Planck Institute for Biological Cybernetics, Tübingen, Germany; 3 Department of Human Perception, Cognition and Action, Max Planck Institute for Biological Cybernetics, Tübingen, Germany; University of Regensburg, Germany

## Abstract

In the Rubber Hand Illusion, the feeling of ownership of a rubber hand displaced from a participant's real occluded hand is evoked by synchronously stroking both hands with paintbrushes. A change of perceived finger location towards the rubber hand (proprioceptive drift) has been reported to correlate with this illusion. To measure the time course of proprioceptive drift during the Rubber Hand Illusion, we regularly interrupted stroking (performed by robot arms) to measure perceived finger location. Measurements were made by projecting a probe dot into the field of view (using a semi-transparent mirror) and asking participants if the dot is to the left or to the right of their invisible hand (Experiment 1) or to adjust the position of the dot to that of their invisible hand (Experiment 2). We varied both the measurement frequency (every 10 s, 40 s, 120 s) and the mode of stroking (synchronous, asynchronous, just vision). Surprisingly, with frequent measurements, proprioceptive drift occurs not only in the synchronous stroking condition but also in the two control conditions (asynchronous stroking, just vision). Proprioceptive drift in the synchronous stroking condition is never higher than in the just vision condition. Only continuous exposure to asynchronous stroking prevents proprioceptive drift and thus replicates the differences in drift reported in the literature. By contrast, complementary subjective ratings (questionnaire) show that the feeling of ownership requires synchronous stroking and is not present in the asynchronous stroking condition. Thus, subjective ratings and drift are dissociated. We conclude that different mechanisms of multisensory integration are responsible for proprioceptive drift and the feeling of ownership. Proprioceptive drift relies on visuoproprioceptive integration alone, a process that is inhibited by asynchronous stroking, the most common control condition in Rubber Hand Illusion experiments. This dissociation implies that conclusions about feelings of ownership cannot be drawn from measuring proprioceptive drift alone.

## Introduction

The Rubber Hand Illusion (RHI) is a tantalizing illusion, where the feeling that a rubber hand belongs to one's body (feeling of ownership) is brought about by stroking a visible rubber hand synchronously to the participant's own occluded hand. In the first work describing this phenomenon, Botvinick and Cohen [1] showed that two types of measures are affected. Participants rated the subjective experience to own the rubber hand high in a questionnaire if stroking was synchronous but not if stroking was asynchronous. In a second experiment, perceived position of the participant's left index finger was measured by an inter-manual reaching task in darkness. A displacement of reaches towards the rubber hand occurred in the case of synchronous stimulation, but not in the case of asynchronous stimulation. This displacement effect has been referred to as *proprioceptive drift* (e.g., [2]). In their study, the magnitude of the proprioceptive drift correlated with the strength of the feeling of ownership reported in the questionnaire.

This proprioceptive drift is usually thought of as a “three-way interaction between vision, touch, and proprioception” [1], in which synchronous stroking (touch) evokes the proprioceptive feeling of the own hand to be displaced towards the seen (visual) rubber hand. Botvinick and Cohen [1] regard proprioceptive drift as evidence in favor of a connectionist model of trimodal processing with all three sensory inputs (vision, proprioception and touch) requiring each other. The model, however, is not further specified. They assert that “the illusion's spurious reconciliation of visual and tactile inputs relies upon a distortion of position sense” (p. 756). Makin, Holmes and Ehrsson [3] propose a more general model of peripersonal space that separates processes of mere visuoproprioceptive integration from those that involve the tactile modality as well; such visuoproprioceptive spatial recalibration effects have been observed in studies with fake or displaced hands even if no touch and feeling of ownership was involved (visual capture of proprioception, e.g., [4]–[7]). Makin et al. [3] propose that “the referral of touch towards the dummy hand […] might in itself be sufficient to induce a (bottom-up) feeling of ownership over the dummy hand” (p. 6) and that visuotactile synchrony of stroking then “further increases the weighting of vision over touch and proprioception in hand position” (p. 6). Sustained stroking may thus gradually increase proprioceptive drift in the RHI as Tsakiris and Haggard [2] report. In a recent review, Tsakiris [8] extends Makin et al. 's [3] model to include a stage where visual inputs are compared to the body state prior to visuoproprioceptive integration to decide whether the multisensory inputs are congruent. The model thus accounts for influences of current body state, such as visual and postural congruency. Most theories of body image stress that body perception is a complex problem and would not advocate a direct connection between different functional domains of using and perceiving our body (e.g., what we perceive to be part of our body vs. where we perceive our limbs to be located). Yet, current models assume that the feeling of ownership enhances existing visuoproprioceptive spatial biases and that synchronous stroking causes proprioceptive drift in the RHI. It is widely assumed that “proprioceptive drifts can be used as a behavioural proxy” [8] to assess the occurrence and also the strength of the subjective feeling to own the rubber hand.

In order to measure the time course of proprioceptive drift, we tested participants in a variant of the RHI paradigm where we interrupted stimulation regularly and recorded participant's perceived finger position. In accordance with existing models of the RHI, we hypothesized that synchronous stroking would lead to a gradual increase in proprioceptive drift. Smaller or no drifts were expected in the asynchronous condition. In Experiment 1, we used a two-alternative forced choice (2AFC) task and an adaptive staircase method to determine finger position. Contrary to our expectation, we observed proprioceptive drifts for both the synchronous and the asynchronous stroking condition. We devised a second experiment to clarify the role of intermittency and synchrony of stroking in causing proprioceptive drifts, using a more immediate (i.e., less time consuming) lateral position adjustment task. In Experiment 2, different frequencies of measurement were compared (every 10 s, 40 s, 120 s). Specifically, we were interested in a possible effect of asynchronous stroking on proprioceptive drift. Experiment 1 suggests such an effect, even if none of the current models would predict it. Just vision of the hand (without stroking) was added as an additional control condition. Based on the results from Experiment 1, we hypothesized that proprioceptive drifts occur for intermittent asynchronous stroking but not for prolonged asynchronous stroking, which was confirmed by the results. We also hypothesized that proprioceptive drift in the just vision condition is the same as synchronous stroking, which was also confirmed. Frequency of measurement during asynchronous stroking is the factor that best accounts for any differences in proprioceptive drift observed.

Our findings show a dissociation of proprioceptive drift and the reported feeling of ownership and suggest that these two phenomena result from different processes of multisensory integration. We argue that prolonged synchronous stroking involves proprioceptive drift in the RHI, but that this drift is already present even without stroking. To the contrary, prolonged asynchronous stroking seems to interfere with visual-proprioceptive integration of the visual location of the rubber hand with the proprioceptive location on the own hand. We argue, that this leads to a less biased percept of the proprioceptive location of one's own hand away from the location of the (visual) rubber hand. Shorter intervals of asynchronous stroking cannot break this integration process so that the drift is still present, despite of the absence of feelings of ownership.

## Materials and Methods

Both Experiment 1 and Experiment 2 were approved by the Ethics Committee of the University Clinics Tübingen, Germany. Informed written consent was obtained from all participants involved in the study.

### Experiment 1: The feeling of hand ownership under frequent measurements

#### Participants

20 paid participants took part in the study (age range: 20–51; median age: 25; 13 female; 17 right-handed, 1 left-handed and 2 ambidextrous, as by self-report). None had previously participated in a RHI study and they were naïve to the purpose of the experiment. 1 had previously heard about the illusion.

#### Experimental Set-Up

**Figure 1:**
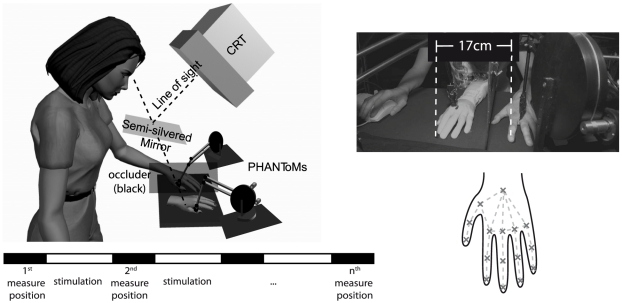
The RHI set-up used in the study. Two PHANToM force-feedback devices with paintbrush endings stroke the subject's occluded hand and the visible rubber hand. Probe dots are projected into the visual field using the CRT and a semi-silvered mirror. Top left: Schematic diagram of the set-up. Top right: photograph of the set-up. Bottom left: time-line of the procedure. Bottom right: patterns of stimulation.

The experiment was conducted using a computer controlled set-up, where two PHANToM force-feedback devices (SensAble Technologies) served as robot arms to stroke both the participant's and the rubber hand with custom-made paintbrush endings (see Fig. 1). A commercially available left rubber hand (10 cm width, 14 cm length from artificial sleeve to fingertip) was placed 17 cm to the right of the participant's real left hand, such that the middle finger of the rubber hand was aligned with the body midline. The real hand was occluded with a matt black cloth. The rubber hand was visible through a 15×15 cm semi-silvered mirror that appeared as a transparent glass if the light below the mirror was switched on (this means that the rubber hand and ca. 7 cm of its lower arm were visible). If the light was switched off, the semi-transparent mirror turned into an opaque mirror, such that the rubber hand was not visible anymore and the reflection of a screen image (CRT monitor mounted on top of the set-up, see Fig. 1) appeared to be in the participant's field of view instead. This technique was used to project a white dot to the left or right of the participant's left hidden index finger tip. The dot was the only thing visible in the dark room during measurement. Throughout the experiment, the participant's hand was never visible. Participants had to judge (forced choice) if the dot was to the left or to the right of perceived position of their unseen index finger (see also “Procedure” below). The dots appeared in the range from 11 cm to the left to 23 cm to the right of the real index finger (with the rubber hand index finger being placed at 17 cm). Answers were given with the right hand (that was also outside the participant's view, Fig. 1, top right). In order to correct for differences in shape and size between the participant's and the rubber hand, prior to the experiment we used a calibration procedure by recording the position of 16 salient points on the real and the rubber hand (i.e., the joints, Fig. 1, bottom right). Strokes were then morphed from the rubber hand to the real hand by linearly interpolating between two neighboring such salient points. Random continuous trajectories of stroking (500 ms–1000 ms strokes) between neighboring points on the hand were generated. In the synchronous condition, corresponding strokes were applied synchronously to both hands. As the paths followed by the paintbrush were random, the stimulation and the velocity of the paintbrush varied with the distance between points and the paths were highly unpredictable. Unpredictability has been linked to the strength of experienced ownership. Experienced subjects reported during piloting that this procedure gives rise to a striking and intense ownership illusion when compared to other procedures, such as manual stroking. In the asynchronous condition, random patterns (spatially and temporally unrelated) were used.

#### Procedure

Participants were divided into 2 groups of 10 each. The first group was tested in the synchronous, the second in the asynchronous stroking condition. Participants were exposed to stroking with measurements of finger proprioception every 10 s for 7 min (timeline: Fig. 1, bottom). For the measurement the light was switched off under the semi-silvered mirror (no vision of the rubber hand or the frame of the monitor or the mirror) and the visual probe dot was projected into the field of view at roughly the same height as the participant's unseen hand and the visible rubber hand. Participants had to respond (forced choice) whether the dot was to the left or to the right of the perceived position of the invisible left index finger of their own hand. The location of the dot changed at every trial according to two alternating simple up-down staircase algorithms (staircase steps: 4 cm and 1 cm) [9]. The staircases move the dot at each step into the opposite direction that the participants report, so that, over time, the dot moves closer to the participant's perceived lateral location of the index finger. Both staircases started at the participant's real index finger position at 0 cm. Before stroking, participants' proprioceptively felt finger position was tested in two pre-tests (one in the dark, one when seeing the rubber hand). At the end of the experiment, participants had to fill out the RHI questionnaire [1], supplemented with a German translation.

### Experiment 2: Frequency of measurement affects proprioceptive drift

#### Participants

Thirty paid participants took part in the study (age range: 18–40; median age: 26; 18 female; 27 right-handed and 3 left-handed, as by self-report). None of them had previously participated in a RHI study and they were naïve to the purpose of the study. Six had previously heard about the illusion.

#### Experimental Set-Up

The set-up was the same as in Experiment 1. To make the stimulation more similar to the manual stimulation used in other studies (e.g., [1]), a 500 ms pause was made between each 500 ms stroke, moving the paintbrush to start the next stroke at a randomly chosen neighboring point. In the synchronous condition, strokes on both hands corresponded in both space and time. In the asynchronous condition, strokes were both temporally out of phase and spatially random.

#### Procedure

Participants were divided into 3 groups of 10. To decrease inter-participant variability, each group was tested in both the synchronous and the asynchronous stroking condition in two blocks that were counter-balanced across participants. Given that the first experiment had shown fast convergence of proprioceptive drifts, visuotactile stimulation was shortened to 2 min in Experiment 2 (most participants experience ownership of the rubber hand within 11 sec [10]). For all groups, perceived finger location in darkness was measured before the onset of stimulation as a baseline measure. In the 1×120 Group, finger proprioception was additionally measured only once after the full length of stimulation, analogous to most RHI studies (e.g., [1]). In the 3×40 Group stroking was interrupted three times (after each 40 sec of stroking) for proprioceptive measurements and in the 12×10 Group such measurements were taken twelve times (after each 10 sec of stroking). This means that the total duration of stimulation is equal in all three groups; the interruption for measurement is added to the total duration of the experiment.

We now used an adjustment task as this is a faster way to assess the perceived finger location compared to the 2 AFC task used in Experiment 1. That is, participants had to adjust the position of a projected dot to match the lateral perceived position of their occluded left index finger in darkness, using the scroll wheel of the computer mouse with the right hand. Only the dot was visible during the adjustment procedure, but not the rubber hand, the frame of the monitor or the mirror. The adjustment was repeated three times each trial and the average of these three adjustments was taken as a data point. The dots could be moved on a horizontal line that ranged from 17 cm to the left to 19 cm to the right of the real index finger position (rubber hand index finger 17 cm to the right). The initial position of these dots was randomized. Relative proprioceptive drift was computed by subtracting the perceived finger position from the pre-test in darkness measured separately for each participant at the beginning of each block.

An additional control condition without stroking (vision only) was added as a third experimental block in the 1×120 Group and the 12×10 Group. No questionnaire ratings were collected in these experiments to avoid biasing participants' perceptual judgments when repeating the measurements in the subsequent blocks of asynchronous and synchronous stroking.

## Results

### Experiment 1: The feeling of hand ownership under frequent measurements

**Figure 2:**
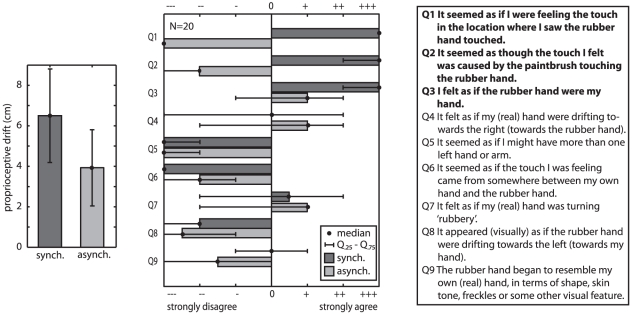
Results from Experiment 1. Left: Proprioceptive drift did not differ significantly between the synchronous and the asynchronous group, mean and s.e.m. N = 20. Right: The questionnaire results confirm that the procedure of interrupting stimulation every 10 s brings about the ownership illusion. Boldface questionnaire items indicate the occurrence of the ownership illusion. In the synchronous condition, questionnaire items Q1 and Q3 are rated significantly positive (p = 0.002; after Bonferroni correction for multiple measures: p = 0.018). Questionnaire item Q2 is nearly significantly positive (one negative reply, p = 0.022; after Bonferroni correction for multiple measures: p = 0.195). Error bars: 25 percentile−75 percentile; N = 20.

As in the study by Botvinick and Cohen [1], the only questionnaire items with a significantly positive rating were items Q1–Q3 in the synchronous stroking group (all three p<0.05 in sign test; Q1 and Q3 after correction for repeated measures, p = 0.018) but not in the asynchronous stroking control group (see Fig. 2). Perceived finger position at the end of the trial was computed by fitting a cumulative Gaussian to the last 12 responses (last 2 min of exposure) to determine the location at which subjects cannot distinguish whether a dot is to the left or to the right of their index finger (50% point of the fitted Gaussian) [11]. A proprioceptive drift towards the rubber hand at the end of the experiment, relative to the pre-test in the dark, was measured in both the synchronous (6.50±2.3 cm, one-tailed t test, t(9) = 2.7, p = 0.013) and the asynchronous condition (3.93±1.88 cm; t(9) = 2.0, p = 0.039; values given in average ± s.e.m.). The difference in proprioceptive drift between the conditions was not significant (two-sample t-test, t(18) = 0.8, p = 0.425).

The results from the questionnaire replicate the results on feelings of ownership of the rubber hand as it has been reported by Botvinick and Cohen [1]. This confirms that the RHI can be induced using the described set-up and particularly the employed procedure with frequent measurements; feelings of ownership only occur with synchronous but not asynchronous stroking. A significant difference in proprioceptive drift between the synchronous and asynchronous conditions, however, could not be replicated; proprioceptive drift occurred in both conditions. This result has two possible explanations. Firstly, frequent measurement could dissociate proprioceptive drift from the feeling of ownership of the rubber hand. Secondly, the experimental procedure (adaptive forced-choice task) could confound or bias the patterns of drift usually reported (i.e., an adaptive procedure may be too slow to capture the rapid perceptual changes).

### Experiment 2: Frequency of measurement affects proprioceptive drift

The result of Experiment 1 suggests that frequent measurement of finger position dissociates proprioceptive drift from the experience to own the rubber hand as reported in a questionnaire in the RHI. In order to verify this, the second experiment measured proprioceptive drift across three groups of participants using different frequencies of measurement (every 10 seconds, 40 seconds and 120 seconds). To exclude any possible biases resulting from the relatively slow experimental adaptive staircase procedure, a more quick and direct position adjustment task was used (see Methods). Additionally, a ‘vision only’ condition was introduced as control condition to assess the importance of tactile stroking.

According to existing accounts of the RHI, it would be expected that synchronous stroking brings about gradual proprioceptive drift that is significantly higher than the drift found for asynchronous stroking or when stroking is omitted in the “vision only” condition. Furthermore, from the existing literature prior to Experiment 1 there was no reason to predict that the frequency of measurement would compromise the effect of drift, which however turns out to be the critical factor.

**Figure 3:**
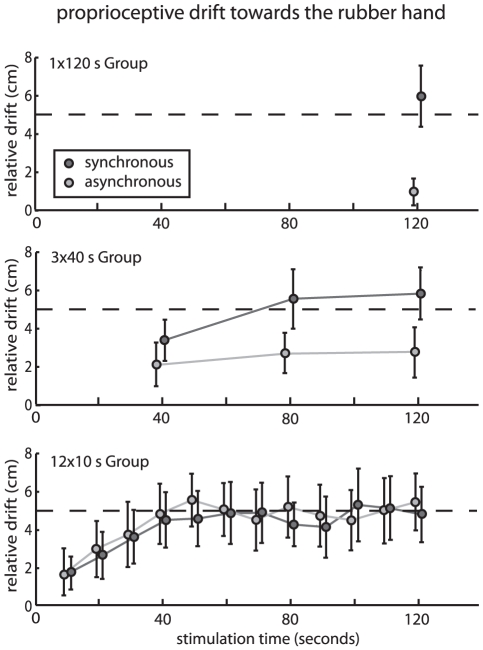
Perceived finger position relative to pre-test across time for the different groups. There is always a drift when synchronous stroking occurs. Surprisingly, there is also proprioceptive drift if asynchronous stroking is interrupted regularly. Error bars: s.e.m. n = 10 in all three groups, dotted line indicates 5 cm line for readability, N = 30.

In all groups, a proprioceptive drift of approximately 5–6 cm was found in the synchronous stroking condition at the end of 2 min of stimulation (Fig. 3), and this drift gradually built up over time. If stimulation was continuous for 120 s, there was no sizeable proprioceptive drift in the asynchronous condition (Fig. 3, top; 0.98±0.69 cm, as opposed to 5.9±1.59 cm in the synchronous condition; all values are mean ± s.e.m.). This replicates earlier results in the literature (e.g., [1]). The drifts observed for intermittent asynchronous stroking, by contrast, followed an unexpected pattern. As the frequency of measurement increased, proprioceptive drift also started to occur in the asynchronous stroking condition. While this drift was still lower than in the synchronous stroking condition in the 3×40 Group (2.75±1.31 cm vs. 5.84±1.38 cm, Fig. 3, middle), the difference in drift between synchronous and asynchronous stroking disappeared in the 12×10 Group (5.45±1.48 cm vs. 4.81±1.44 cm, Fig. 3, bottom) as if the frequency of measurement modulated the drift in the asynchronous condition, but not the synchronous condition. A 3×2 mixed ANOVA with drift as dependent variable, group as between subjects factor (1×120 s, 3×40 s, 12×10 s), and mode of stroking as within subjects factor (synchronous, asynchronous) confirmed that the interaction between the frequency of measurement (between groups) and the mode of stroking (within groups) was significant (F(2,27) = 3.9, p = 0.031). There was also a main effect of the mode of stroking, but not of the frequency of measurement (F(1,27) = 8.9, p = 0.006, F(2,27) = 0.5, p = 0.635). The main effect of stroking was due to the 12×10 Group and the 3×40 Group, but not the 12×10 Group (paired-sample t tests t(9) = 2.7, p = 0.024, t(9) = 2.5, p = 0.034, t(9) = 0.6, p = 0.592). The equal levels in proprioceptive drift for both synchronous and asynchronous stroking in the 12×10 Group confirmed the dissociation between visuotactile synchrony and the proprioceptive drift effects under frequent measurement that were already found in Experiment 1.

A possible explanation for this surprising result is that asynchronous stroking interferes with the integration process that leads to effects of vision on proprioception in any situation of visuoproprioceptive conflict (i.e., seeing a hand displaced from its real location, e.g. [5]). The longer such asynchronous stroking lasts, the stronger is the evidence against the “unity assumption” [12], that is the assumption that the proprioceptive and the visual sensations belong together. This unity assumption is a pre-requisite for visuoproprioceptive integration to occur. If vision and proprioception would not be sensed as belonging together, no integration of the sensations would occur and thus no drift would be observed.

**Figure 4:**
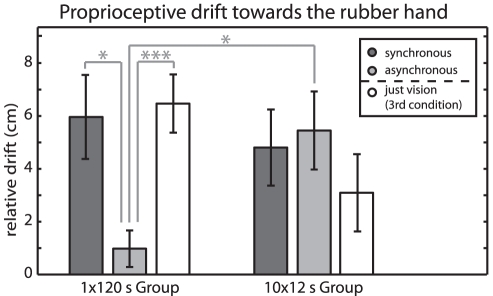
Proprioceptive drift for synchronous stroking, asynchronous stroking and just vision. Perceived finger position relative to pre-test at the end of the block for the 1×120 Group and the 12×10 Group. The drift measured for just vision is the same as when the drift measured for synchronous stroking. Error bars: s.e.m. n = 10 in both groups, N = 20.

To further test the hypothesis that asynchronous stroking is necessary to diminish the effect of drift by breaking the unity assumption, we measured proprioceptive drift in a condition without any stroking (i.e., only vision of the rubber hand). If asynchronous stroking is necessary to diminish the drift effect at the end of 2 minutes of tactile stimulation, it should still be present in the vision only condition. We tested the vision only condition in both the 1×120 Group and the 12×10 Group. Figure 4 shows the results from this additional condition; in agreement with the hypothesis outlined above, the drift observed in this condition was as strong as the drift in the synchronous stroking condition in both groups and stronger than the drift in the asynchronous stroking condition in the 1×120 group (6.47±1.10 cm; 12×10 s: 3.09±1.46 cm). A 2×3 mixed ANOVA with group (1×120, 12×10) as between subjects factor and mode of presentation (synchronous, asynchronous, just vision) as within subjects factor showed no main effect of the group (F(1,18) = 0, p = 0.990) or the mode of presentation (F(2,36) = 2.4, p = 0.109), but a significant interaction between frequency of measurement and mode of presentation (F(2,36) = 7.8, p = 0.002). Two one-way repeated measures ANOVAs with mode of presentation (synchronous, asynchronous, just vision) as factor showed a significant difference in proprioceptive drift within the 1×120 group (F(2,18) = 7.6, p = 0.004) but not in the 12×10 group (F(2,18) = 1.7, p = 0.22). The interaction is due to the asynchronous stroking condition; additional paired sample t-tests showed a significant difference between the asynchronous and just vision conditions (t(9) = 4.8, p<0.001) but no difference between the synchronous and just vision conditions (t(9) = 0.3, p = 0.76). This supports the hypothesis that differences in drift reported in earlier studies are driven by asynchronous stroking. Within participants, the drift magnitude in the synchronous and the just vision condition was correlated (p = 0.030, Pearson's correlation coefficient r = 0.49; data pooled from 1×120 Group and 12×10 Group), further supporting the hypothesis that proprioceptive drift in the RHI and proprioceptive drift in the just vision condition are one and the same phenomenon. The apparently lower levels of drift in the just vision condition of the 12×10 group is not statistically significant (compared to just vision in 1×120 group: t(18) = 1.75, p = 0.097).

In Experiment 2, no questionnaire ratings were recorded in-between the different conditions (synchronous, asynchronous, vision only) to avoid biasing the participants' responses in subsequent conditions and also because two of the three relevant questionnaire items (Q1 and Q2) refer to the localization of touch and are thus not applicable to the vision only condition. However, anecdotally, the vast majority of participants reported a strong feeling of ownership in the synchronous condition, but not in the asynchronous condition or vision only condition in the debriefing after completion of the experiment, irrespective of the frequency of measurement. In agreement with this result, Longo et al. [13] have reported that just vision of a rubber hand does not elicit the feeling of ownership.

There were no order effects for the order of synchronous or asynchronous exposure in Experiment 2. A 3×2 mixed ANOVA with group (1×120, 3×40, 12×10) as between subjects factor and mode of presentation (synchronous, asynchronous) as within subjects factor showed no main effect of the group (F(2,27) = 0.4, p = 0.635) the mode of presentation (F(1,27) = 1.3, p = 0.260) or their interaction (F(2,27) = 0.1, p = 0.919). As just vision was always the third condition, it is confounded with order, so it is impossible to test for order effects during this block.

In Experiment 2, we observed proprioceptive drift of equal magnitudes not only if the participant's hand and the rubber hand were stroked synchronously, as it would be expected from current accounts of the RHI, but also when no stroking was applied and even if asynchronous stroking was interrupted every 10 s. The only condition that does not lead to such a drift and that thus accounts for all differences in drift levels observed is prolonged asynchronous stroking (Fig. 4). This result cannot be an artifact of the frequent measurement procedure (response bias). The presentation of dots was random from an interval centered on the real position of the participant's hand. If the initial position of dots would bias the outcome of the adjustment procedure, this bias should, if at all, bias the results towards the position of the real index finger. Response bias can therefore not explain the proprioceptive drift towards the rubber hand in the 12×10 s group. The drift results from the exposure to the visually displaced rubber hand. For these reasons, we propose the possibility that asynchrony is interpreted as evidence against the “unity assumption” [12] necessary for cross-modal integration. In this interpretation, asynchronous stroking counters visuoproprioceptive integration, independent of the experience of ownership of the rubber hand, i.e., the two measures are dissociated.

## Discussion

### Proprioceptive drift and the ownership illusion do not go hand in hand

It is known that visual-proprioceptive integration can bias perceived limb position in ways that are similar to the proprioceptive drift observed in the RHI without the feeling of ownership of the fake hand (e.g., [6]). So what is new about our result? Firstly, we show that drift in the prolonged asynchronous stroking condition is not only significantly smaller than the drift in the synchronous stroking condition, as expected. It is also significantly smaller than drift in the vision only condition and intermittent asynchronous stroking condition (Fig. 4). This suggests that prolonged visuotactile asynchrony has an effect on the perception of hand location in the presence of a visuoproprioceptive conflict (less bias towards the visual position of the hand). Even though the possibility that differences in drift may be due to asynchronous stroking has been mentioned previously [14], this is not usually considered to be the case.

Secondly, we directly compared the synchronous and the vision only condition and could thus show that visuoproprioceptive integration and proprioceptive drift in the RHI are equally strong and correlated within participants. Proprioceptive drift gradually increases whenever it occurs, even without feeling ownership of the rubber hand. This indicates that synchronous stroking may not induce any proprioceptive drift additional to the drift resulting from visuoproprioceptive integration. As the subjects did not report experience of ownership in the just vision condition, there is dissociation between the two phenomena, where low drift requires prolonged asynchronous stroking, and the feeling of owning the rubber hand requires visuotactile synchronous stroking.

Some findings from earlier studies support our conclusion that proprioceptive drift in the RHI may not be caused by synchronous stroking, but rather that its lack may be caused by asynchronous stroking in the control condition. Tsakiris and Haggard's [2] results on the time course of proprioceptive drift appear to involve a certain amount of drift also in the asynchronous condition. This is in line with our results from the 3×40 Group (Fig. 3, middle) that tests a similar frequency of measurement (every 40 s vs. every 60 s). Durgin, Evans, Dunphy, Klostermann and Simmons [15] have shown that proprioceptive drift and the feeling of ownership occur in a situation that did not involve tactile stimulation at all, but instead used visual “stimulation” with a laser, which calls into question the role of tactile stimulation by stroking in the RHI. However, this visual laser-stimulation still brings about the feeling of ownership and thus does not pose a direct challenge to the presumed link between the feeling of ownership and the proprioceptive drift; the drift measured for laser-stimulation could still have been causally related to the processes that make participants experience ownership over the artificial hand.

### Underlying Mechanisms

Existing accounts of the RHI assume processes of multisensory integration where visuotactile synchrony provides information in favor of the unity assumption, which causes both the feeling of ownership of the rubber hand and the proprioceptive drift towards the rubber hand. Thus, proprioceptive drift during the RHI is explained as a “three-way interaction between vision, touch, and proprioception” [1]. That is, vision and proprioception are merged (leading to drift and the feeling of ownership) only or stronger in case there is congruent tactile-visual interaction from synchronous stroking. Botvinick and Cohen [1] propose a “constraint-satisfaction process operating between vision, touch and proprioception […] structured by the correlations normally holding among these modalities” (p. 756) as a common cause of the drift and the feeling of ownership. If there was such a direct link, synchronous tactile stimulation should increase the drift found for just vision or intermittent asynchronous stroking, as these latter conditions do not involve feeling of ownership of the rubber hand.

The alternative explanation offered by our results is that differences in proprioceptive drift, such as in Fig. 3 (top), are driven by asynchronous stroking in the control condition that seems to provide evidence against the “unity assumption” [12] and thus weakens visuoproprioceptive integration (visual capture of proprioception, e.g., [5]), rather than synchronous stroking causing it. The interruptions we introduced to measure the proprioceptive drift have the unexpected effect to also interrupt the accumulation of evidence against the unity assumption (note that it is not absolute duration of stroking that modulates the drift in the asynchronous condition; the absolute duration of stroking is kept constant across groups). If the proprioceptive signal is weakened over time (participants keep their hand still), there is also a simple explanation for the gradual increase in proprioceptive drift. It has been reported that the proprioception of hand position drifts towards the body midline when the hand is kept still (e.g., [16], [17]), which supports the outlined possibility.

This possibility afforded by our results is counter-intuitive at first; it seems plausible that the experienced acquisition of a displaced limb (feeling of ownership) should involve a spatial update. However, a closer look at the literature in cognitive neuroscience reveals abundant evidence that for different cognitive and behavioral domains, the body is perceived and used differently (e.g., [14], [18]). Our perceived body boundaries do not always linearly map to how we move, coordinate and locate our body in space. The proposal here is that the sensation of owning a rubber hand does not cause any automatic update of the perceived position of the body in space; that the processes underlying the proprioceptive drift are independent of the visuotactile integration that causes the feeling of ownership. From our results it cannot be ruled out that the visuotactile integration may be a pre-requisite of the feeling of ownership, but the feeling of ownership does not itself influence the spatial update, nor does the spatial update itself cause the feeling of ownership. The common comparison of synchronous and asynchronous stroking leads to the erroneous impression that the differences in proprioceptive drift and the feeling of ownership are directly linked. Our results provide direct evidence in favor of a dissociation of the two effects and their underlying mechanisms.

Makin et al. 's [3] model of peripersonal space can be reconciled with this account if some modifications are applied. The authors assume that visuotactile synchrony provides positive feedback on existing processes of visuoproprioceptive integration. Under this assumption, the model cannot explain our results. However, the weakening of visuoproprioceptive integration by asynchronous stroking can be interpreted as negative feedback on the visual weight in their model of peripersonal space. With this modification, their model can account for our results. The same holds true for Tsakiris' [8] preliminary model, which extends Makin et al. 's model by introducing a module that compares visual information with the current state of the body prior to multisensory integration. Such a revision would mean to implement the counter-intuitive result suggested by our study, i.e., that there is no direct connection between proprioceptive drift and the synchronous stroking that brings about the feeling of ownership of the rubber hand.

We cannot without further evidence assume that the results we found for visual judgments of perceived hand position generalize to other techniques to measure perceived finger location, like inter-manual reaches. We can only hypothesize that asynchronous stroking in the most common control condition for the RHI has a general negative effects on the visual capture of proprioception.

### Using Proprioceptive Drift as a Proxy for the Ownership Illusion

Proprioceptive drift is thought of as a stable behavioral correlate of the feeling of ownership, as reflected for instance in Kammers et al. 's assessment that “numerous studies have demonstrated that perceptual judgments are affected by the RHI” ([14], p. 205). It has been reproduced in a variety of extensions and variations of the original RHI experiment, using both inter-manual reaches (e.g., [9], [14], [15], [19]–[25]) and visual estimations of perceived finger position in space (e.g., [2], [14], [22], [26]–[33]). A similar change in perceived body location has been shown in a full-body variant of the illusion [34]. Several studies have confirmed that the strength of the feeling of ownership correlates with the magnitude of proprioceptive drift (e.g., [1], [27], [28]).

At first glance, a direct causal link, i.e., that both measures are driven by synchronous stroking, seems the most parsimonious explanation for this correlation. Even though theories of body image often stress the multi-facetted nature of human body perception (e.g., [27]), models and experiments frequently implement simplified assumptions. Given that the correlation between proprioceptive drift and the subjective feeling of rubber hand ownership appears to be so robust, it has been proposed that “proprioceptive drifts can be used as a behavioural proxy” [8] of the experience to own the rubber hand. It is habitual to refer interchangeably to the feeling of ownership and the proprioceptive drift as “the” RHI and interpret results in terms of a causal link between the two or to use the proprioceptive drift as a measure of the RHI (e.g., [2], [14], [19], [25], [26], [30]–[32]).

If there is no direct causal connection between the feeling of ownership and the proprioceptive drift, as we claim, what is the source of the correlation between proprioceptive drift and the feeling of ownership previously observed? A more remote common cause can explain correlation in the absence of direct causal links. For instance, more “visual” participants may be more susceptible to both visual capture of proprioception and to the feeling that they own the rubber hand. Alternatively, proprioceptive drift may be a pre-requisite for the occurrence of the feeling of ownership, even if the feeling of ownership does not influence the proprioceptive drift. In either case, the practice of using proprioceptive drift as a proxy for the feeling of ownership would be problematic. If it was admissible to assess the feeling of ownership by proxy of the proprioceptive drift, we could conclude from our results that body ownership is modulated by the frequency of measurement in asynchronous stroking (Fig. 3). Only the complementary questionnaire ratings (Fig. 2) show that this is not true.

Doubts similar to ours about a direct causal link between the feeling of ownership and the proprioceptive drift as a proxy have been expressed previously (cf. Makin et al. [3] for an overview). Observing visual capture of hand proprioception, Holmes at al. [6] warned that “reaching or proprioceptive biases are not reliable objective measures of the rubber hand illusion itself” and that the underlying processes may be “causally unrelated” (p. 700). However, so far, there have been no alternative explanations for the differences in proprioceptive drift reliably reported and no direct comparison of synchronous stroking and no stroking has been made to call into question that synchronous stroking at least adds to the effect.

### How to measure the RHI

The doubts that our results cast on the validity of using proprioceptive drift as a proxy to measure the intensity of feeling of ownership in the RHI leads to a more general question of how the RHI can be quantified.

Everyone who has experienced the subjective feeling of body ownership in the RHI will confirm that it is most fascinating. This experience is the true “illusion” in the RHI and, arguably, it is the aspect of the illusion that has earned it its popular interest. Scientifically speaking, however, this dimension is the most difficult to tackle. Beside questionnaires, as in the original study [1], vividness ratings have been used to directly quantify the feeling of ownership (e.g., [35], [36]). To date, the most systematic enquiry into the subjective dimension of the RHI has been performed by Longo et al. [27], using a 27-item questionnaire on a large participant population to identify different phenomenological components in the illusion. Longo et al. [27] argue that the fact that “components of experiences are selectively related to proprioceptive biases attests to [their method's] validity” (p. 995). It is important to recognize the value of such a psychometric approach as a tool in its own right; their psychometric results stand out as a novel approach to address complex questions of subjective experience in perception research, even if the “behavioral grounding” is here called into question.

Beside proprioceptive drift, a number of other behavioral and physiological measures have been found to be affected in the RHI experiment, including skin conductance response (SCR) to threat [20], [35], [37]–[39], skin temperature [36], rate of self-recognition [40] and cross-modal congruency [33]. While it is interesting in itself that synchronous stroking affects these variables, it seems particularly attractive to interpret changes in these variables as an indication of how strongly a subject feels ownership of the rubber hand. Indirect measurement would allow bypassing problems inherent in the direct quantification of subjective experience (e.g., suggestion, variability due to beliefs and top-down influences). The results presented in this paper demonstrate the dangers of such an endeavor. The question that remains is: When a behavioral or physiological measure has been shown to correlate with the strength of felt ownership, if and under which circumstances can it be used as a measure or proxy for how strongly hand ownership is experienced? The more plausibly it can be argued that the same mechanism brings about both the feeling of ownership and the behavioral correlate in question, the stronger can one be seen as an indicator of the other. For instance, SCR has been shown to be closely associated with changes in subjective affective states across many contexts, not just the RHI, which makes it plausible that there may be a link. However, as long as the generative mechanisms of the RHI are not fully understood, there can be no certainty about causal links. It is thus advisable to be careful with using behavioral or physiological correlates as exclusive measure or proxy for the RHI (supplementary subjective measures, such as vividness ratings, can be used). Findings should be interpreted conservatively, taking into consideration the possibility of correlation without causation.

The same principal limitations apply to neural and neuro-behavioral correlates of the illusion (e.g.,[10], [22], [31], [32], [41]–[43]); a correlation is only meaningful in so far as it can be argued to be causally linked to the phenomenon in question. For instance, Ehrsson et al. [10] found activity in multisensory areas (premotor cortex and intraparietal cortex) using functional magnetic resonance imaging. They could not only show that vividness ratings correlate with the neural activity but also that the onset of activity in these areas corresponds to the reported onset of the subjective feeling of ownership. These kinds of additional findings indicate that the activity is probably not due to visuoproprioceptive integration alone. On the other hand, Tsakiris et al. [32] found a correlation between activity in the right insular cortex (positron emission topography) and the RHI in a study that exclusively measured proprioceptive drift. This study has been interpreted as evidence that the insula cortex plays a key role in producing the feeling of ownership (e.g., [8]). Had Tsakiris et al. [32] recorded complementary vividness ratings and analyzed the relation between neural activity and different processes of multisensory integration, their findings would be a much more solid basis for conclusions about the neural correlates of subjectively experienced body ownership.

A different approach to study the RHI is to test it in patients with impaired body image. On the one hand, schizophrenia [44], [45] and anorexia nervosa [46] have been found to bias the experience of the illusion. On the other hand, inducing the illusion has been reported to modulate tactile extinction [47] and even the experience of limb ownership in amputees [20], [48]. Such research on the RHI in the context of neuro- or psychopathology bears great potential for therapeutic approaches in the spirit of the “mirror box” therapy for phantom limbs [49]. Yet, if the mechanisms of body ownership remain unknown, such an approach may raise more questions than it answers from the perspective of basic cognitive neuroscience, even if it serves well for therapeutic intervention.

## Conclusion

In the RHI, a drift in the proprioceptively sensed hand position has been reported to correlate with the subjective feeling of owning the rubber hand (e.g., [1]). This drift has been readily accepted and widely used as a proxy for the subjective feeling of ownership. Our results show that asynchronous stroking in the control condition has a negative effect on this drift and may indeed be responsible for changes in proprioceptive drift previously reported in the literature. The processes of spatial updating of the body in space and the subjective feeling of body ownership are dissociated in our paradigm, which suggests that separate mechanisms of multisensory integration underlie the two effects (spatial update and feeling of ownership). The effects of asynchronous stroking on different measures used in RHI experiments has so far not been explicitly investigated. Current models of the RHI need to be revised to explain this result. Furthermore, our results suggest that the practice of using proprioceptive drift to assess feelings of ownership is problematic.
